# Whole genome deep sequencing analysis of cell-free DNA in samples with low tumour content

**DOI:** 10.1186/s12885-021-09160-1

**Published:** 2022-01-20

**Authors:** Devika Ganesamoorthy, Alan James Robertson, Wenhan Chen, Michael B. Hall, Minh Duc Cao, Kaltin Ferguson, Sunil R. Lakhani, Katia Nones, Peter T. Simpson, Lachlan J. M. Coin

**Affiliations:** 1 Institute for Molecular Bioscience, University of Queensland, St Lucia, Brisbane, Australia; 2 Department of Clinical Pathology, The University of Melbourne, Parkville, Melbourne, Australia; 3 Centre for Clinical Research, Faculty of Medicine, The University of Queensland, Herston, Brisbane, Australia; 4 Pathology Queensland, The Royal Brisbane & Women’s Hospital, Herston, Brisbane, Australia; 5 QIMR Berghofer Medical Research Institute, Herston, Brisbane, Australia; 6 Department of Microbiology and Immunology, The University of Melbourne, Parkville, Melbourne, Australia; 7 Department of Infectious Disease, Imperial College London, London, UK

**Keywords:** Cell-free DNA, Cell-free tumour DNA, Somatic mutations, Mutational signatures

## Abstract

**Background:**

Circulating cell-free DNA (cfDNA) in the plasma of cancer patients contains cell-free tumour DNA (ctDNA) derived from tumour cells and it has been widely recognized as a non-invasive source of tumour DNA for diagnosis and prognosis of cancer. Molecular profiling of ctDNA is often performed using targeted sequencing or low-coverage whole genome sequencing (WGS) to identify tumour specific somatic mutations or somatic copy number aberrations (sCNAs). However, these approaches cannot efficiently detect all tumour-derived genomic changes in ctDNA.

**Methods:**

We performed WGS analysis of cfDNA from 4 breast cancer patients and 2 patients with benign tumours. We sequenced matched germline DNA for all 6 patients and tumour samples from the breast cancer patients. All samples were sequenced on Illumina HiSeqXTen sequencing platform and achieved approximately 30x, 60x and 100x coverage on germline, tumour and plasma DNA samples, respectively.

**Results:**

The mutational burden of the plasma samples (1.44 somatic mutations/Mb of genome) was higher than the matched tumour samples. However, 90% of high confidence somatic cfDNA variants were not detected in matched tumour samples and were found to comprise two background plasma mutational signatures. In contrast, cfDNA from the di-nucleosome fraction (300 bp–350 bp) had much higher proportion (30%) of variants shared with tumour. Despite high coverage sequencing we were unable to detect sCNAs in plasma samples.

**Conclusions:**

Deep sequencing analysis of plasma samples revealed higher fraction of unique somatic mutations in plasma samples, which were not detected in matched tumour samples. Sequencing of di-nucleosome bound cfDNA fragments may increase recovery of tumour mutations from plasma.

**Supplementary Information:**

The online version contains supplementary material available at 10.1186/s12885-021-09160-1.

## Background

Cell-free DNA (cfDNA) is an emerging non-invasive biomarker for diagnosis and prognosis of various acute and chronic disorders. cfDNA has been detected in many body fluids, including plasma, serum, urine and cerebrospinal fluid [1]. cfDNA is predominantly of hematopoietic origin [2], however recent studies have showed release of cfDNA from other organs and tissues into the extracellular compartments [3–5]. The connection of cfDNA with several tissues and organs in the body makes it an attractive non-invasive biomarker for various diseases including cancer.

Cell-free tumour DNA (ctDNA) derived from cancerous cells can be detected in blood [6] and it has provided new avenues for non-invasive detection and monitoring of cancer [7]. Tumour-specific alterations such as somatic copy number aberrations (sCNAs) and single nucleotide variants (SNVs) have been detected in the plasma of cancer patients [8]. ctDNA has been detected in both early and late stage tumours [9] and the utility of ctDNA as a biomarker has been assessed for various cancer types with promising results [10]. The levels of ctDNA can be used as an early diagnostic marker and to monitor changes during therapy [11–13].

Currently the gold standard approach for tumour diagnosis involves biopsy sampling. However, the invasive nature of the biopsy sampling process restricts its use. It is not feasible for frequent sampling; the size and the location of the tumour also imposes limitations. Moreover, a biopsy only samples part of the tumour, hence, only represents a fraction of the possible heterogeneity observed in many tumours. ctDNA on the other hand can be obtained by a single blood draw allowing for multiple sampling. Also, as ctDNA is derived from various tumour clones and sites, it provides a comprehensive representation of the tumour heterogeneity in the patient [5]. These features make them an ideal biomarker for cancer diagnosis and monitoring.

Levels of ctDNA can vary between different cancer types and often early stage cancers have very low levels of ctDNA in plasma [9], making it difficult to measure. To enable accurate detection of ctDNA, targeted approaches such as quantitative PCR for specific gene mutations or copy number changes associated with cancer are widely used [14–16]. Targeted sequencing approaches using gene panels or exome panels have been utilised to enable testing of more targets in cfDNA [17, 18].

To date, most sequencing approaches on cfDNA for the detection of tumour-derived genomic alterations have been based on either targeted sequencing approaches or low-coverage whole genome sequencing (WGS) approaches. Higher sequencing coverage achievable via targeted approaches have facilitated detection of cancer related mutations even in samples with low ctDNA [19]. However targeted sequencing approaches cannot capture all genomic changes, such as structural rearrangements. Low-coverage WGS approaches are widely utilised to assess CNAs in ctDNA [20–23]. The size of the CNAs and the levels of ctDNA in the sample affects the efficiency of this approach [24]. In contrast to targeted sequencing, single nucleotide mutations cannot be accurately detected using low-coverage WGS approach.

The cost associated with sequencing approaches has mainly hindered the use of high-coverage WGS approaches on cfDNA. However, simultaneous detection of gene mutations and CNAs in cfDNA can be achieved by WGS approaches [8, 25]. In this study, we aim to explore the utility of high-coverage WGS of cfDNA in cancer diagnosis. We performed high-coverage (~100X coverage) WGS analysis of cfDNA from patients with breast tumours and patients with benign tumours. We identified a large fraction of somatic mutations in cfDNA samples not detected in matched tumour samples and identified specific somatic mutational signatures in these samples. We also explored the differences in fragment size distribution in cfDNA samples.

## Methods

### Sample collection

Four patients with breast cancer (1084, 1249, 1494 and 1524) and 2 patients with benign tumours (065 and 098) were included in this study. Tumour characteristics of these samples are provided in Supplementary Table 1. These patients were recruited by the Brisbane Breast Bank [26], which was approved by the Human Research Ethics Committee at the University of Queensland (Project ID: 2005000785) and the Royal Brisbane and Women’s Hospital (Ref. 2005/022). Tumour tissue samples were collected during surgery and blood samples were collected prior to the surgery from these patients. Tumour samples from benign tumour patients were not sequenced.

EDTA blood tubes were processed on the same day (between 1.5 to 5 h) of collection. Blood samples were centrifuged at 3000 rpm for 10 min to separate blood cells and plasma. The buffy coat was stored at -20^ο^C for germline DNA extraction. Plasma aliquots were re-centrifuged at 13000 rpm for 10 min and the plasma was stored at -80^ο^C for plasma cfDNA extraction. Tissue samples from tumours were snap frozen in liquid nitrogen and stored in − 80 degrees freezer.

### DNA extraction

Plasma cfDNA was extracted using Circulating Cell free Nucleic Acid kit (Qiagen) according to manufacturer’s instructions. Germline DNA from Buffy coat was extracted using the QIAamp DNA Blood Mini kit (Qiagen) and tumour DNA from tissue samples were extracted using the AllPrep Universal kit (Qiagen) according to manufacturer’s instructions.

### Library preparation

Libraries for sequencing were prepared using the TruSeq Nano HT Kit (Illumina) according to manufacturer’s instructions with minor modifications for plasma cfDNA samples. Briefly germline and tumour DNA samples (100 ng input DNA) were fragmented to 350 bp and a size selection step was performed after the end repair process to remove small fragments. However, due to the nature of plasma cfDNA (20 ng input DNA), which consists of short DNA fragments, fragmentation and size selection were omitted during the library preparation. The rest of the process were similar for all DNA samples and prepared according to the manufacturer’s instructions.

Libraries were sequenced on Illumina HiSeqXTen and 150 bp paired-end sequencing was performed. Samples were sequenced at varying coverage for each sample type; plasma cfDNA samples were sequenced in 4 lanes of HiSeqXTen per sample, tumour samples were sequenced in 2 lanes per sample and germline DNA samples were sequenced in 1 lane to achieve 120X, 60X and 30X coverage respectively.

### Pre-processing of sequencing reads

FastQC [27] was used to assess the quality of the FastQ files. Trimmomatic (v0.32) [28] was used to trim Illumina adapter sequences and low quality bases (base quality less than 30) in both ends of the read. Also reads less than 35 bp in length were discarded. Base quality was low towards the end of the read, therefore all reads were trimmed to 145 bp regardless of quality using fastx_trimmer (FASTX-Toolkit [29]).

Sequence reads were aligned to human genome hg19 reference version using BWA MEM [30]. Samtools (v1.3) [31] was used to filter out supplementary alignments. MarkDuplicates option in Picard tools [32] was used to identify duplicated reads. Scripts used for processing of sequencing reads are provided in https://github.com/Devika1/Plasma_HiSeqXTen.

### Somatic variant analysis

Somatic single nucleotide variant (SNV) detection was performed using VarScan2 (version 2.4.4) [33] for both tumour and plasma samples. Samtools (v1.10) [31] mpileup with default settings (except minimum mapping quality of 2) was used to generate the input for Varscan2 variant calling. Samtools mpileup, by default considers overlapping reads and counts them only once and ignores duplicated reads in the read counts. VarScan2 with *somatic* option was used with default settings, except 0.01 frequency was used for ‘min-var-freq’ option. The output was then processed with *processSomatic* option with default settings, except for --min-tumor-freq 0.01 and --max-normal-freq 0.00 to identify high confidence somatic variants. These high confidence variants were further filtered with *fpfilter* option with default settings except for --min-var-freq 0.01 to remove false positive variants. Bam-readcount (version 0.8.0, https://github.com/genome/bam-readcount) was used to calculate readcounts, mapping quality and base quality at the variant positions and this information was used in the fpfilter to determine false positive variants. Output from fpfilter were filtered further using the following thresholds to identify high confidence somatic variants:

i) At least 10x coverage for germline and tumour sample
ii) At least 5 reads supporting the variant allele in tumour sample
iii) 0 reads in germline for the variant allele

### Variant annotation

Somatic variants identified from VarScan2 were annotated using Annovar [34] and hg19 human databases were used for annotation. Somatic variants which were shared between tumour and plasma samples were identified using custom awk scripts (provided in https://github.com/Devika1/Plasma_HiSeqXTen).

### Somatic reads enrichment

Reads supporting somatic variants were used for downstream analysis and these reads were extracted using a java package JAPSA (https://github.com/mdcao/japsa). The somatic reads extraction tool was deployed using script name *jsa.hts.aareads*. Filtered somatic output from VarScan2 (to provide position of somatic variants) and aligned bam file was used as input to extract reads containing the somatic variant (script provided in https://github.com/Devika1/Plasma_HiSeqXTen).

### Mutational signature analysis

Mutational Patterns [35] was used to identify mutational signatures from the somatic mutation data. Somatic SNVs (specifically single base substitutions) from plasma and tumour samples were used for mutational signature analysis. Mutational Patterns R package was used for analysis (https://github.com/UMCUGenetics/MutationalPatterns). De novo mutational signature extraction was performed using Non-negative Matrix Factorization (NMF). Contributions of known COSMIC mutational signatures (version 2) (https://cancer.sanger.ac.uk/cosmic/signatures_v2) for SNVs were determined from the mutational profiles of each samples and this information was used to determine the mutational process.

### Somatic CNAs analysis

Somatic copy number aberration (CNA) analysis was performed using IchorCNA [18]. Readcounts for IchorCNA analysis were generated using HMMcopy readcounter option (https://github.com/shahcompbio/hmmcopy_utils). Readcounts were generated for 1 Mb window and reads with mapping quality of greater than or equal to 20 were used. CNAs in both tumour and plasma samples were assessed using IchorCNA with matched germline sample as normal control. For plasma samples, tumour content was expected to be low, therefore estimated tumour fractions of 5, 1, 0.5 and 0.1% were used and ploidy was set to diploid. However, for tumour samples estimated tumour fractions of 50, 60, 70, 80, 90% were used and ploidy was set to 2 and 3.

### Fragment size distribution

Samtools (v1.10) was used to extract reads less than 2000 bp insert size. A customised python script (provided in https://github.com/Devika1/Plasma_HiSeqXTen) was used to compute the number of reads per fragment size. We calculate the number of reads in each category (all reads, reads which have a somatic mutation, reads with a somatic mutation which is shared with tumour, reads with a somatic mutation which is unique to plasma) as a function of the length of the read, x. We calculate the ratio of shared to unique mutations for all reads with length less than or equal to x bp, as well as the ratio of unique to shared for all reads with length greater than x bp.

## Results

### Generation of high coverage cell-free DNA sequencing data

Matched germline, tumour and plasma samples were sequenced on Illumina HiSeqXTen and Table 1 summarizes the sequencing output achieved per sample. Sequencing coverage (Table 1) varied between samples, however expected sequencing coverage of 30X and 60X were achieved for germline and tumour DNA samples, respectively. Sequencing yield for plasma DNA samples was less than expected, nevertheless an average of 100X sequencing coverage was achieved for plasma DNA samples, representing one of the few high-coverage WGS datasets for cfDNA.

**Table 1:** HiSeqXTen Sequencing output per sample

Specimen Type	Sample^a^	Number of reads	Sequencing Yield (Mb)	% Bases > = Q30	% Duplicated reads	Sequencing Coverage^b^
Germline DNA	1084_N0^c^	885,589,680	132,838	83.88	15.82	35
	1249_N0	927,175,866	138,149	87.61	37.67	28
	1494_N0	830,442,242	123,736	85.08	27.42	29
	1524_N0	937,319,184	139,661	86.33	25.54	34
	065_N0^c^	897,176,584	134,576	85.10	8.30	40
	098_N0^c^	1,014,190,632	152,129	86.09	11.31	44
Tumour DNA	1084_T0	1,785,014,202	265,967	82.32	31.09	58
	1249_ T0	1,919,380,364	285,987	83.99	26.70	67
	1494_T0	1,833,108,412	273,133	80.67	22.14	66
	1524_T0	1,819,092,474	271,044	83.42	24.43	65
Plasma DNA	1084_P0	3,978,736,468	592,832	88.76	25.10	97
	1249_P0	3,742,076,682	557,569	87.92	28.30	82
	1494_P0	3,703,572,042	551,832	89.22	29.78	83
	1524_P0	3,993,601,472	595,047	89.77	26.60	93
	065_P0^c^	4,247,100,536	637,065	85.56	12.77	112
	098_P0^c^	4,145,998,174	621,900	84.70	10.96	116

^a^*N0* germline, *T0* tumour, *P0* plasma

^b^Sequencing coverage was estimated using Isaac [36] provided by the sequencing provider; duplicated reads and overlapping bases are excluded for the coverage calculation

^c^Sequenced in a separate batch

### Somatic variant analysis

VarScan2 [33] was used to detect somatic single nucleotide variation (SNV) in plasma and tumour samples. DNA samples from blood buffy coat were used as germline controls to exclude germline variants in plasma and tumour samples. Somatic variants were filtered as described in Methods to identify high confidence somatic variants. Table 2 summarizes the number of somatic SNVs detected in plasma and matched tumour samples and the number of shared SNVs observed between matched tumour and plasma samples. All coding mutations in both plasma and tumour samples are provided in Additional File 1.

**Table 2:** Summary of somatic variants in sequenced samples

Sample*	Number of somatic SNVs	Number of Mutations/ Mb	Annotation of Somatic SNVs			Number of shared somatic SNVs	% of shared SNVs
			Exonic		Intronic		
			Total	Non-synonymous			
**1084_P0**	4056	1.35	37	25	854	228	5.6%
**1084_T0**	6070	2.02	77	59	1950		3.8%
**1249_P0**	4142	1.38	39	27	897	387	9.3%
**1249_T0**	1120	0.37	13	10	288		34.6%
**1494_P0**	3433	1.14	22	11	783	262	7.6%
**1494_T0**	1271	0.42	18	11	392		20.6%
**1524_P0**	4771	1.59	39	23	1090	281	5.9%
**1524_T0**	2841	0.95	37	23	840		9.9%
**065_P0**	3857	1.29	38	24	897	–	–
**098_P0**	5637	1.88	47	28	1356	–	–

* *T0* tumour, *P0* plasma, * Patients with breast cancer - 1084, 1249, 1494 and 1524; patients with benign tumours - 065 and 098

We detected a similar number of somatic variants in all 6 plasma samples (average 4316 SNVs), whereas the number of somatic variants varied between different tumour samples (range 1120–6070 SNVs), which could be due to the inherent heterogeneity in breast cancer genomes, as well as the variable tumour purity of individual samples (sample 1249 had very low tumour purity of 14%, whereas other tumours were greater than 65%; Refer Supplementary Table 1). Approximately 4–35% of the somatic variants observed in tumour samples were detected in matched plasma samples, however these shared variants accounted for only 6–10% of the total somatic variants detected in plasma. The majority of somatic variants were unique to each plasma and tumour sample (Supplementary Fig. 1).

### Variant allele frequency

We analysed the variant allele frequency (i.e. sequence coverage for variant alleles) of somatic variants in tumour and plasma samples. We also assessed the distribution of the variant allele frequency of variants which were shared between matched tumour and plasma samples and unique variants which were only present in either tumour or plasma samples.

**Fig. 1:**
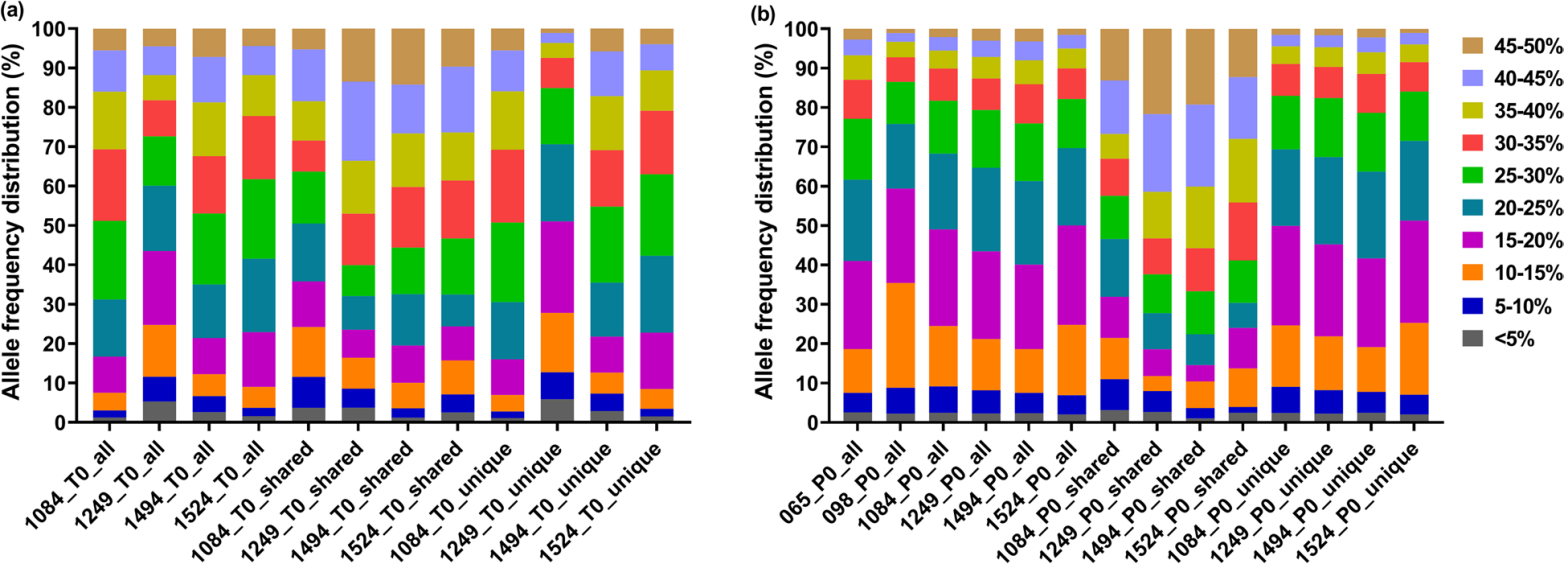
Allele frequency distribution of somatic variants in (**a**) tumour and (**b**) plasma samples. All refers to all somatic variants in the sample; shared refers to variants which were shared between matched tumour and plasma samples and unique refers to variants which were only present in either tumour or plasma samples. Samples 065 and 098 were from benign tumour patients and other samples were from breast cancer patients

Variant allele frequency distribution of all somatic variants in tumour samples (Fig. 1a) varied between samples possibly due to the tumour purity of the samples (Supplementary Table 1). Similarly, variant allele frequency of shared and unique somatic variants in tumour samples differed between samples. Between 16 and 51% of the unique variants in tumour samples had less than 20% variant allele frequency. This could explain why only 4 to 35% of the tumour mutations (Table 2) were detected in plasma samples.

Variant allele frequency distribution of all somatic variants in plasma samples (Fig. 1b) from cancer patients (samples 1084, 1249, 1494 and 1524) showed less variation between samples compared to tumour samples. Between 40 and 59% of all somatic variants in plasma samples had less than 20% variant allele frequency. Less than 30% of the shared variants in plasma samples had less than 20% allele frequency indicating that majority of the shared variants had higher variant allele frequency. Approximately 50% of the unique variants in plasma samples had allele frequency less than 20%.

### Annotation of somatic variants

We performed gene analysis on somatic variants for both plasma and tumour samples and identified missense and nonsense mutations in several coding genes (Refer to Additional File 1). We also detected mutations in cancer associated genes reported in COSMIC Cancer Gene Census (CGC) [37] for both plasma and tumour samples. Table 3 summarises the number of mutations observed in Cosmic CGC genes (exact genomic mutation changes are provided in Additional File 2). Mutations in multiple breast cancer driver genes such as *NOTCH1, PIK3CA,* and *TP53* were detected in tumour samples, however these mutations were not detected in matched plasma samples. Mutations in COSMIC CGC genes such as *BCLAF1, MUC4,* and *RGPD3* were observed in multiple plasma samples. There were no mutations in Cosmic CGC which were shared between matched plasma and tumour samples.

**Table 3:** Mutations observed in genes reported in COSMIC Cancer Gene Census (CGC)

Sample	Missense mutations		Nonsense mutations	
	Number of SNVs	Cosmic CGC genes	Number of SNVs	Cosmic CGC genes
1084_P0	2	BCLAF1, MUC4	–	–
1084_T0	3	CARD11, CCR4, KDSR	–	–
1249_P0	2	BCLAF1^a^, MUC4, RGPD3^b^	–	–
1249_T0	1	COL3A1	–	–
1494_P0	–	–	–	–
1494_T0	2	KAT6A, PPARG	1	TP53
1524_P0	3	BCLAF1^a^, KMT2C, MUC4	–	–
1524_T0	4	MUC4, NOTCH1, PIK3CA, TP53	–	–
065_P0	–	–	–	–
098_P0	2	BCLAF1^a^, RGPD3^b^	–	–

^a^The exact mutation for BCLAF1 (c.G2243T: p.R748L)

^b^The exact mutation for RGPD3 (c.T2811G: p.S937R)

### Somatic signatures

Different mutational processes create characteristic mutational signatures on the genome. Hence, patterns of somatic mutations can indicate the mutational processes which have been active in a tumour. Large-scale analyses of cancer genome data across various cancer types have revealed recurrent mutational signatures [38, 39]. We used Mutational Patterns [35] to extract these mutational signatures in our samples.

**Fig. 2:**
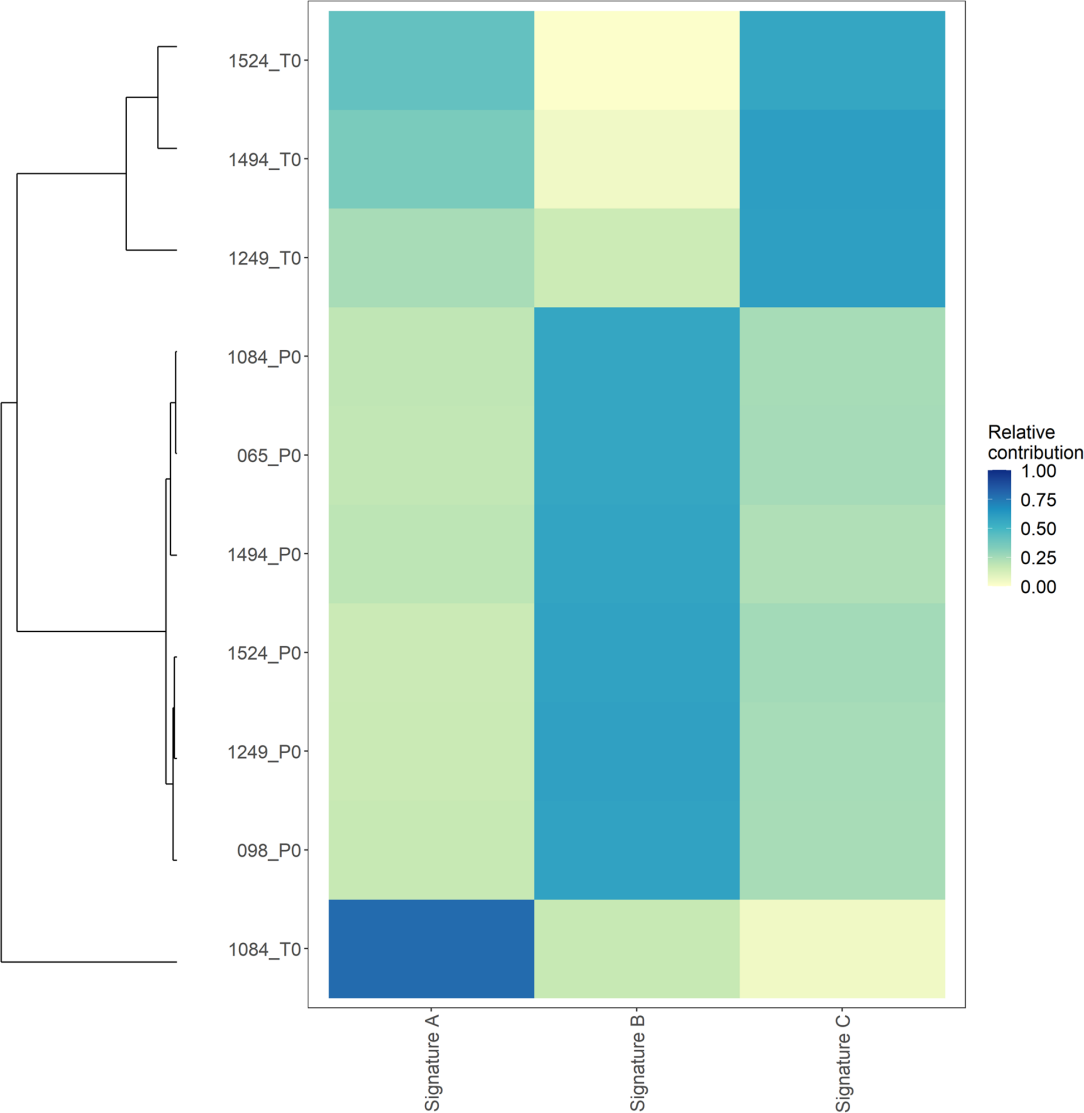
Relative contribution of de novo mutational signatures in plasma and tumour samples. P0 – denotes plasma samples and T0 – denotes tumour samples

Mutational changes due to C > T and T > C were predominant in both plasma and tumour samples (Supplementary Fig. 2 and Supplementary Fig. 3). We performed de-novo mutational signature detection using non-negative matrix factorization (NMF). We extracted mutational signatures and compared their relative contribution in plasma and tumour samples (Fig. 2). Based on the extracted signatures, it was evident that the mutational profiles were different between plasma and tumour samples. Signature A and Signature C was prominent in tumour samples whereas Signature B was prominent in all plasma samples (Supplementary Fig. 4).

We compared the extracted mutational profiles of plasma and tumour samples to the known COSMIC mutational signatures (version 2). Supplementary Table 2 shows the Cosine similarity [35] between the extracted signatures and COSMIC signatures. Signature A was similar to COSMIC signatures 3 and 8 (which are commonly seen in Breast cancer [40] and signature 5 (which is common to all cancers [41]), whereas Signature C was similar to signature 5. On the other hand, Signature B, which was enriched in all plasma samples, was similar to signatures 5 (common to all cancers) and 16 (found in liver cancer [42]).

**Fig. 3:**
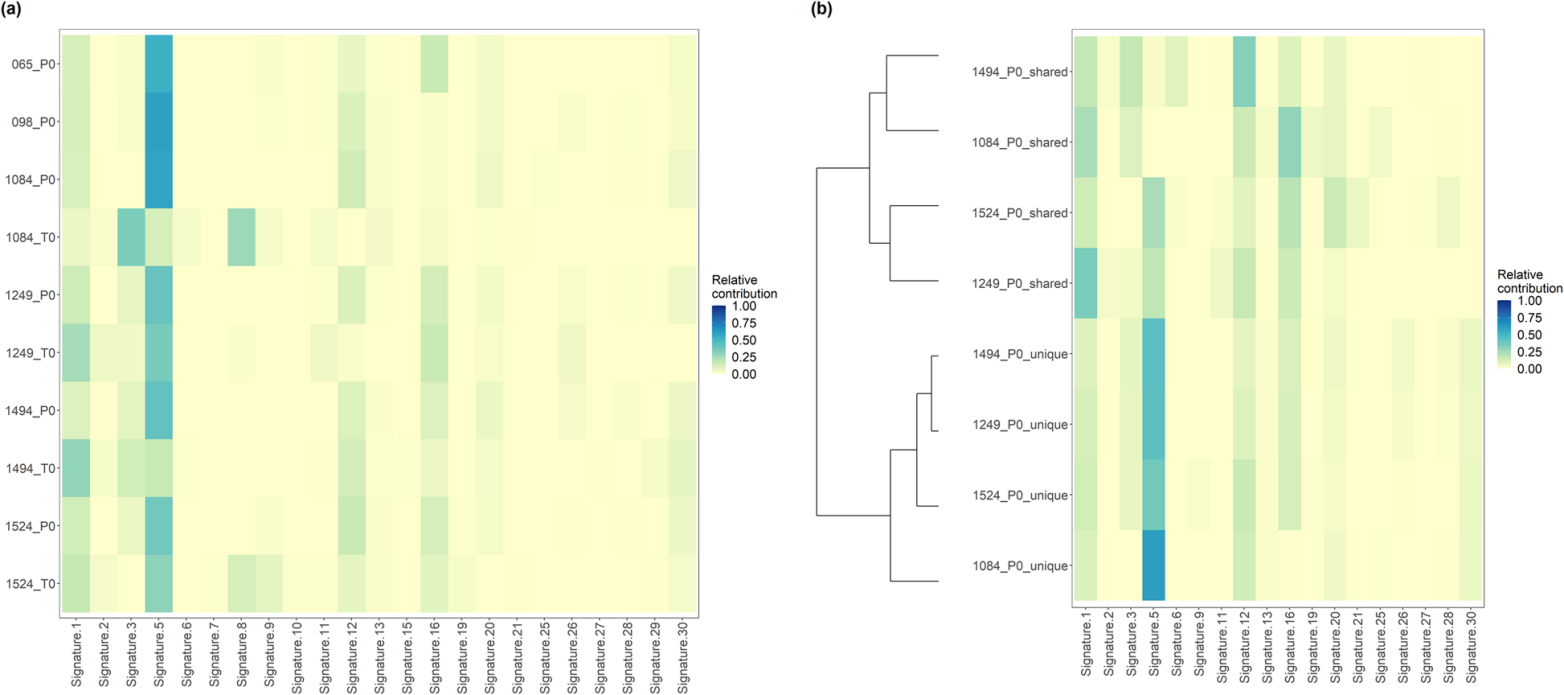
Heatmap showing the relative contribution of COSMIC mutational signature for (**a**) all somatic mutations in plasma and tumour samples; (**b**) somatic mutations which were shared with matched tumour and mutations which were unique to plasma samples. P0 – denotes plasma samples and T0 – denotes tumour samples. Samples 065 and 098 were from benign tumour patients and other samples were from cancer patients

We compared the mutational profiles of plasma and tumour samples directly with known COSMIC mutational signatures (version 2). Figure 3a shows the COSMIC mutational signatures observed in plasma and tumour samples. Supplementary Table 3 shows the Cosine similarity for the mutational profiles and COSMIC signatures. Signature 5 was observed in all plasma and tumour samples and notably had higher contribution in plasma samples compared to matched tumour samples. Signature 5 has been found in all cancer types and the aetiology is unknown [41]. Signature 16 was also present in all plasma samples. Signature 16 has been found in liver cancer and the aetiology is unknown [42]. Signatures 1, 3 and 8 were found in multiple tumour samples. Signature 3 has been found in breast, ovarian, and pancreatic cancers, and associated with failure of DNA double-strand break-repair by homologous recombination and signature 8 is found in breast cancer and medulloblastoma and the aetiology is unknown [40]. There were not any significant differences in COSMIC mutational signature contribution between plasma samples from benign tumour patients (065_P0 and 098_P0) and cancer patients. The relative contribution of COSMIC mutational signatures in plasma and tumour samples is shown in Supplementary Fig. 5.

We assessed the mutational signatures in plasma samples for somatic mutations which were shared with matched tumour and somatic mutations which were unique to plasma (i.e. not detected in matched tumour) (Fig. 3b). The contribution of Signature 5 was more prominent in unique mutations compared to shared mutations. Signatures which were prominent in tumour samples, such as signatures 1, 3 and 8 (Fig. 3a) were also observed in plasma shared mutations. Comparison with unique mutations in plasma and tumour samples revealed that Signature 5 was present in all samples (Supplementary Fig. 6). However, Signature 5 was more prominent in plasma unique mutations compared to tumour unique mutations. This indicates that the unique mutations in plasma samples contain mutations which are different from tumour and possibly acquired from somatic changes in other tissues.

### Analysis of somatic CNAs

**Fig. 4:**
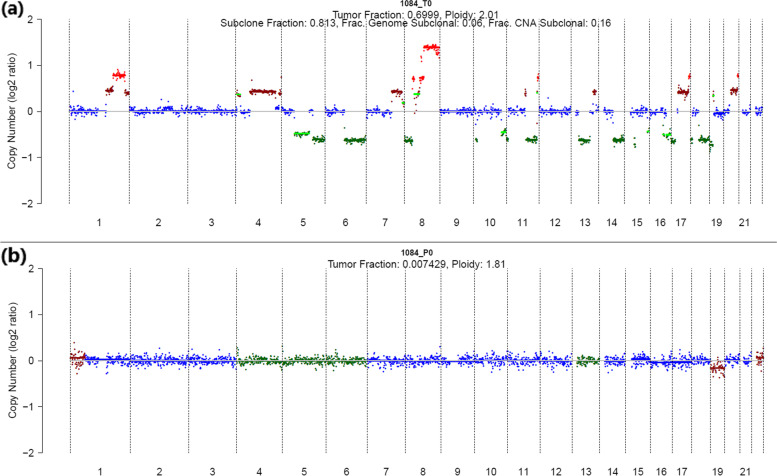
Somatic CNAs detected in patient 1084 (**a**) tumour and (**b**) plasma samples. Copy number across chromosome 1 to 22 are plotted. The colour of the data points denotes copy number; dark green - 1 copy, blue - 2 copy, brown – 3 copy and red – 4 copy. Light green horizontal line represents a subclonal prediction

Somatic CNAs in both plasma and tumour samples was detected using IchorCNA [18]. For both plasma and tumour samples matched germline DNA was used as control. Various somatic CNAs were detected in all 4 tumour samples and tumour fraction determined by IchorCNA was 70, 13, 41 and 33% for 1084_T0, 1249_T0, 1494_T0 and 1524_T0 samples, respectively. However, somatic CNAs were not detected in any of the plasma samples and estimated tumour fractions were less than 1% for all 6 plasma samples. Figure 4 shows the somatic CNAs detected in sample 1084 tumour and matched plasma sample. IchorCNA plots for all other samples are provided in Supplementary Fig. 7.

We also used all 6 plasma samples as a combined normal panel for plasma sample analysis; however, it did not identify any somatic CNAs in plasma samples. We used our in-house tool sCNASeq [43] to detect somatic CNAs, but it also failed to detect any somatic CNAs in plasma samples. This is likely due to the low tumour content in the plasma samples and the analysis approaches are not sensitive enough to analyse low tumour content samples despite high sequencing coverage in the samples.

### Fragment size analysis

**Fig. 5:**
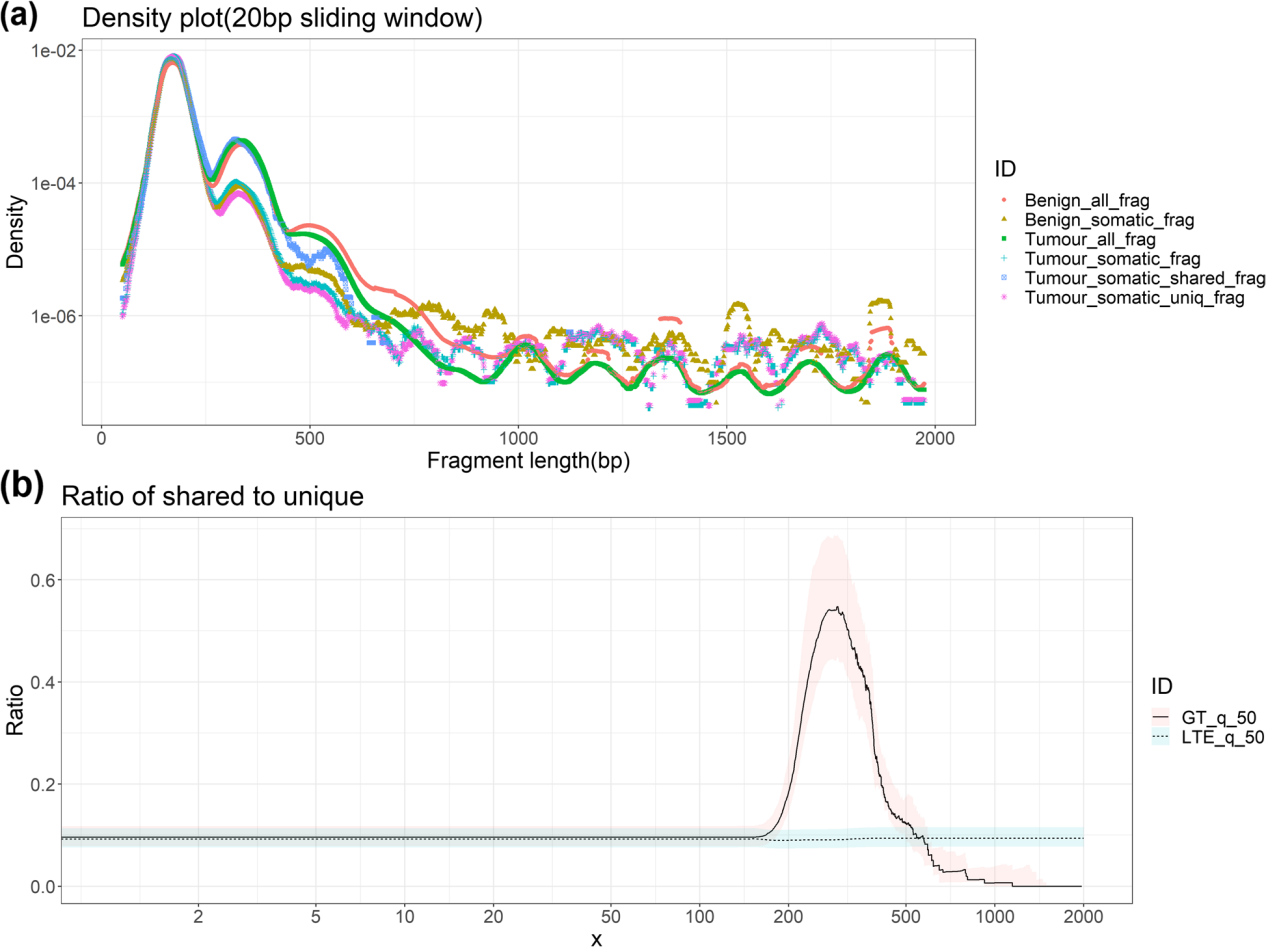
(**a**) Cell-free DNA fragment length distribution for all reads and somatic reads in both tumour patients and benign tumour patients plotted as cumulative density plot. (Tumour_all_frag – all reads from 4 cancer patients; Tumour_somatic_frag – all somatic reads from 4 cancer patients; Tumour_somatic_shared_frag - all shared somatic reads (i.e. reads from somatic variants which were present in matched tumour) from 4 cancer patients; Tumour_somatic_uniq_frag – all unique somatic reads (i.e. reads from somatic variants which were not present in matched tumour) from 4 cancer patients; Benign_all_frag – all reads from 2 benign tumour patients; Benign_somatic_frag – all somatic reads from 2 benign tumour patients) (**b**) Fragments less than and greater than x bp are compared between shared somatic reads and unique somatic reads in cancer patients. The plot shows the interquartile range, and the lines refers to 50% quantile, GT – greater than x and LTE – less than or equal to X. The reads are combined from all 4 cancer patients

Plasma DNA fragments exhibit a unique fragment length profile due to the nucleosome positioning; hence the majority of the cfDNA fragments are approximately 166 bp (mono-nucleosome size) and multiples thereof. We assessed the fragment length distribution of all reads with less than 2000 bp insert size and observed the expected fragment size distribution pattern for cfDNA (Supplementary Fig. 8). We extracted all somatic reads which contain a somatic SNV (Refer to Methods) and assessed the fragment length distribution of somatic reads. We further grouped the somatic reads based on the somatic SNVs which were shared or not shared (i.e., unique) with matched tumour samples and explored the differences in fragment length between all reads, somatic reads, somatic shared reads and somatic unique reads for both cancer patients and benign tumour patients. We noticed differences in fragment length profile between somatic reads and all reads in all plasma samples (Fig. 5a). There was not any difference in somatic fragment length distribution between cancer patients and benign tumour patients.

Fragment length comparison between somatic shared reads and somatic unique reads in tumour patients revealed that tumour-derived fragments (i.e., somatic shared reads) were enriched in fragments 300 bp -350 bp compared to somatic unique reads (Fig. 5b). We found a higher proportion of somatic shared reads were enriched in fragments within the di- nucleosome peak compared to somatic unique reads (Fig. 5a and b). This suggests that some tumour-derived fragments could be longer and less fragmented. Higher enrichment of tumour-derived shared fragments in the longer size range indicates that it could be feasible to selectively enrich fragments between 300 and 400 bp to enrich for tumour-derived fragments in plasma samples.

## Discussion

Currently ctDNA analysis are often performed using targeted sequencing of small panels of genes or known hotspot mutations in key cancer genes. Low-coverage WGS analysis of cfDNA is often performed for detection of somatic CNAs. To-date, only a handful of studies have performed high-coverage WGS (20-50x coverage) of cfDNA for tumour analysis [8, 25]. Use of high-coverage WGS for cfDNA analysis is mainly constrained due to the high cost of sequencing. However, it has the potential to discover all somatic changes in cfDNA samples. In this study, we performed deep sequencing analysis of cfDNA samples to explore its utility for detection of tumour-derived somatic changes in samples with low tumour content and to improve our understanding on the biology of cfDNA. The sequencing data generated in this study is one of the highest coverage cfDNA sequencing data with matched tumour sequencing data. This could be a valuable resource for researchers working in non-invasive diagnostic approaches to develop novel analytical methods and to understand the biological characteristics of cfDNA.

Despite the high sequencing coverage, we only detected less than 10% of somatic SNVs in plasma which were shared with matched tumour. One of the main reasons for this is the low-tumour content in the plasma samples. The CNA analysis estimated that the tumour content in the samples to be less than 1%. Theoretically with 100X coverage, a variant with 1% allele frequency would only have 1 variant supporting read, which is not sufficient to reliably call the variant allele. Variation in sequence coverage across the genome could detect these low frequency variants. It was evident from the allele frequency distribution analysis that greater than 70% of the shared variants in the plasma samples had greater than 20% variant allele frequency, indicating that only high frequency tumour variants were detected in the matched plasma.

We performed a stringent variant filtering for somatic variant analysis to reduce false positives. Only 32–45% of the tumour mutations were detected in plasma samples. Variant allele frequency distribution indicated that the variants which were not detected in plasma were mostly low frequency variants. However, some of the key tumour driver mutations such as *TP53* and *PICK3CA* had high variant allele frequency in tumour, yet these were not detected in plasma. This could be due to the detection limit of the somatic variant caller we have used and may be resolved by other somatic mutation detection tools such as Mutect [44] and LoFreq [45], however their detection sensitivity for low frequency variants needs to be explored. Though, VarScan2 combined with strict variant filtering as performed in our study is recommended for detecting low frequency mutations [46].

Greater than 90% of the somatic mutations detected in plasma samples were unique to plasma samples. Most studies to date on plasma somatic variant analysis have only used targeted sequencing and they have also reported mutations in plasma samples which were not detected in matched tumour samples (approximately 50–90% of variants were not shared with matched tumours) [18, 47]. One of the possible reasons for this divergence could be the bias in tumour sampling and associated tumour heterogeneity, where only a fraction of the tumour is sampled and analysed. Some of the somatic mutations identified in the plasma could have been present in the tumour, but it might not been present in the precise piece of tumour sequenced, due to sampling variations. Also, the presence of metastatic tumours could also contribute to somatic variations in the plasma.

The mutational burden of the plasma samples (average 1.44 somatic mutations/Mb of genome) was higher than the tumour samples (average of 0.94 somatic mutations/Mb of genome). High-coverage targeted sequencing of gene panels on plasma of controls and cancer patients have revealed mutations due to clonal hematopoiesis [48, 49] and often most of these mutations were detected in matched blood samples in low-frequency. Clonal hematopoiesis describes the expansion of a clonal population of hematopoietic stem cells regardless of disease state [50, 51]. These contribute to low-frequency somatic clones in blood, which are released into plasma and then detected in plasma cfDNA. Although, in this study we used matched blood samples to exclude germline variants, it is likely some of the low-frequency variants in the blood samples were not detected due to relatively low-coverage (30x) of germline DNA samples compared to plasma DNA samples. Hence, it is possible that some of the somatic mutations detected in the plasma samples in our study could have been due to clonal hematopoiesis.

The other possible explanation for higher mutational burden in plasma samples, could be due to clonal somatic changes in germline tissues. Multiple somatic variants were identified in normal tissues suggesting a macroscopic clonal expansions in normal tissues leading to somatic mosaicism [52, 53]. Given that plasma cfDNA contains DNA from various organs and tissues [4, 5], any somatic changes in these cells could be detected in plasma. Hence it is possible that some of these somatic variants in plasma are derived from other tissues. However, methylation or transcriptomic or nucleosome positioning analysis needs to be performed to ascertain what fraction of somatic variants are derived from other tissues.

Mutational signature analysis on plasma samples revealed higher contribution of signature 5, which has been found in all cancer types and most cancer samples [38, 39]. Furthermore, unique somatic mutations in plasma samples had higher contribution of signature 5 compared to shared somatic mutations indicating that these mutations are distinct to tumour-derived mutations. It has been reported that Signature 5 is driven by the loss of *FHIT* gene [41]. Depletion of *FHIT* causes replication stress-induced DNA double-strand breaks and defects in replication fork progression and prevents activation of DNA damage response [54]. It is likely that the majority of somatic mutations in plasma are likely due to the result of replication errors and lack of DNA damage responses.

Tumour-derived somatic CNAs are detected in plasma samples of cancer patients using low-coverage WGS [21, 22]. However, samples with high tumour content are often used in these analyses. Plasma samples in our study had low tumour content, hence detection of somatic CNAs was not feasible. Most somatic CNA detection tools use large number of normal cfDNA samples as a reference panel. Although IchorCNA could use single matched germline sample as the normal control, performance of CNA detection is improved with large normal panels [18]. Due to the lack of large normal cfDNA high coverage WGS data, it was not feasible to detect somatic CNAs in low tumour content samples, despite high sequencing coverage.

Cell-free DNA fragments commonly show a prominent peak at 166 bp, due to nucleosome positioning and suggesting apoptosis based DNA fragmentation [5, 55]. Size distribution of tumour-derived DNA have revealed enrichment in fragment sizes between 90 and 150 bp for multiple tumour types [56] and longer ctDNA fragments (> 1000 bp) are also enriched in some cancer types [57]. However, we did not detect any enrichment in tumour-derived fragments in < 150 bp, this is likely due to the low tumour content in our plasma DNA samples. Mouliere et al. (2018) have demonstrated that low ctDNA samples have less fragments below 150 bp compared to samples with high ctDNA content [56]. We detected an enrichment in somatic shared fragments in 300 bp - 350 bp, indicating higher fraction of tumour-derived fragments in cfDNA di-nucleosome peak. Higher enrichment in the longer fragment range of 300 bp - 350 bp indicates that it could be feasible to selectively enrich these longer size fragments to improve the detection sensitivity of tumour-derived fragments in plasma samples with low tumour content.

It was evident from our analysis, that despite the high sequencing coverage of 100X in plasma samples, it was not sufficient to detect all somatic changes in low-tumour content samples. We believe the sequencing dataset generated in this study will be valuable for other researchers to develop novel analytical tools to improve the detection sensitivity for somatic changes and to further explore the characteristics of cfDNA.

## Conclusion

In summary, high-coverage WGS analysis of cfDNA samples revealed a large fraction of unique somatic variants in plasma, which are likely derived from somatic clonal changes in germline tissues. Characteristics of these somatic mutations are different to the tumour-derived somatic mutations in plasma samples. High-coverage WGS analysis did not detect all tumour-derived somatic changes in samples with low-tumour content. Nevertheless, it did improve our understanding on the biology of cfDNA and this study provides a valuable high-coverage WGS dataset of cfDNA to facilitate further research.

## Supplementary Information

**Additional file 1.** Supplementary File-List of coding mutations.

**Additional file 2.** Supplementary File-List of mutations in COSMIC Cancer Gene Census genes.

**Additional file 3.** Supplementary Information-Supplementary Tables and Figures.

## Data Availability

The datasets supporting the conclusions of this article are available in the European Genome Archive (EGA) repository, under data accession number EGAD00001006869 (https://ega-archive.org/datasets/EGAD00001006869).

## Abbreviations

cfDNA: Cell-free DNA
ctDNA: Circulating tumour DNA
WGS: Whole genome sequencing
SNVs: Single nucleotide variants
CNAs: Somatic copy number aberrations
