# Supplementary material for: Whole genome deep sequencing analysis of cell-free DNA in samples with low tumour content
Source: BMC Cancer. 2022 Jan 20;22:85. doi: 10.1186/s12885-021-09160-1 (PMC8772083; doi:10.1186/s12885-021-09160-1)
Supplement: Supplementary file 3 — Additional file 3. Supplementary Information-Supplementary Tables and Figures. [file 12885_2021_9160_MOESM3_ESM.docx]

**Supplementary Information:**

**Supplementary Table 1:** Characteristics of the tumour samples used in the study

| **Sample_ID** | **1084** | **1249** | **1494** | **1524** | **065** | **098** |
| --- | --- | --- | --- | --- | --- | --- |
| **Type** | Invasive & In Situ | Invasive & In Situ | Invasive & In Situ | Invasive & In Situ | Benign | Benign |
| **Subtype** | Luminal | Luminal | Luminal | Luminal | Fibroadenoma | Fibroadenoma |
| **Invasive Diagnosis** | Invasive Ductal Carcinoma | Invasive Ductal Carcinoma | Invasive Ductal Carcinoma | Mixed Ductolobular carcinoma | NA | NA |
| **Grade** | 2 | 3 | 3 | 3 | NA | NA |
| **ER** | Positive | Positive | Positive | Positive | NA | NA |
| **PR** | Positive | Positive | Positive | Positive | NA | NA |
| **HER2 (IHC)** | 1 | 0 | 0 | 0 | NA | NA |
| **HER2 (CISH)** | 2 | 2.1 | 3.4 | 3.2 | NA | NA |
| **Tumour Purity estimate (SNP array)** | 78.06% | 13.74% | 81.42% | 65.76% | NA | NA |
| **Tumour Size** | 40mm | 25mm | 35mm | 14mm | NA | NA |

NA – Not applicable


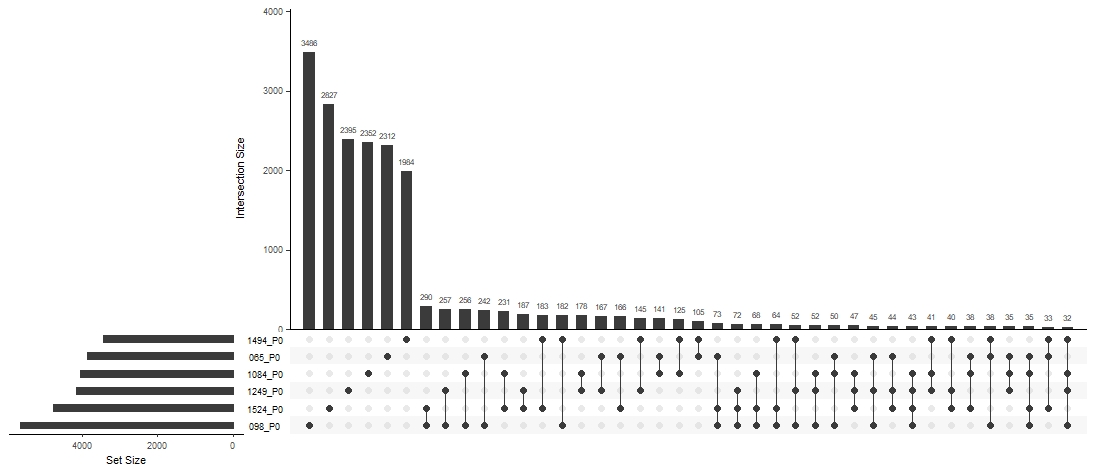


(a)


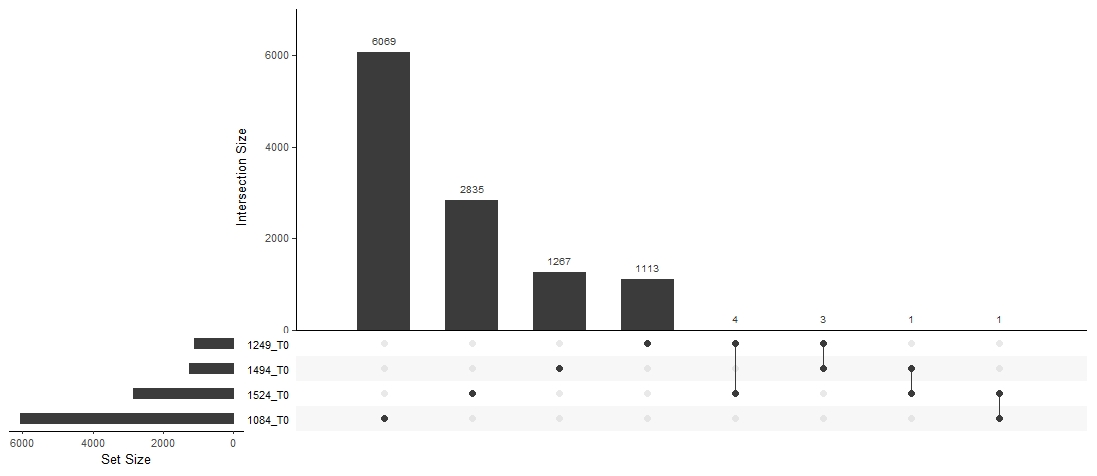
 **Supplementary Figure 1:** Upset plots shows the overlap between somatic mutations in (a) plasma and (b) tumour samples. Samples 065 and 098 were from benign tumour patients and other samples were from cancer patients.

(b)


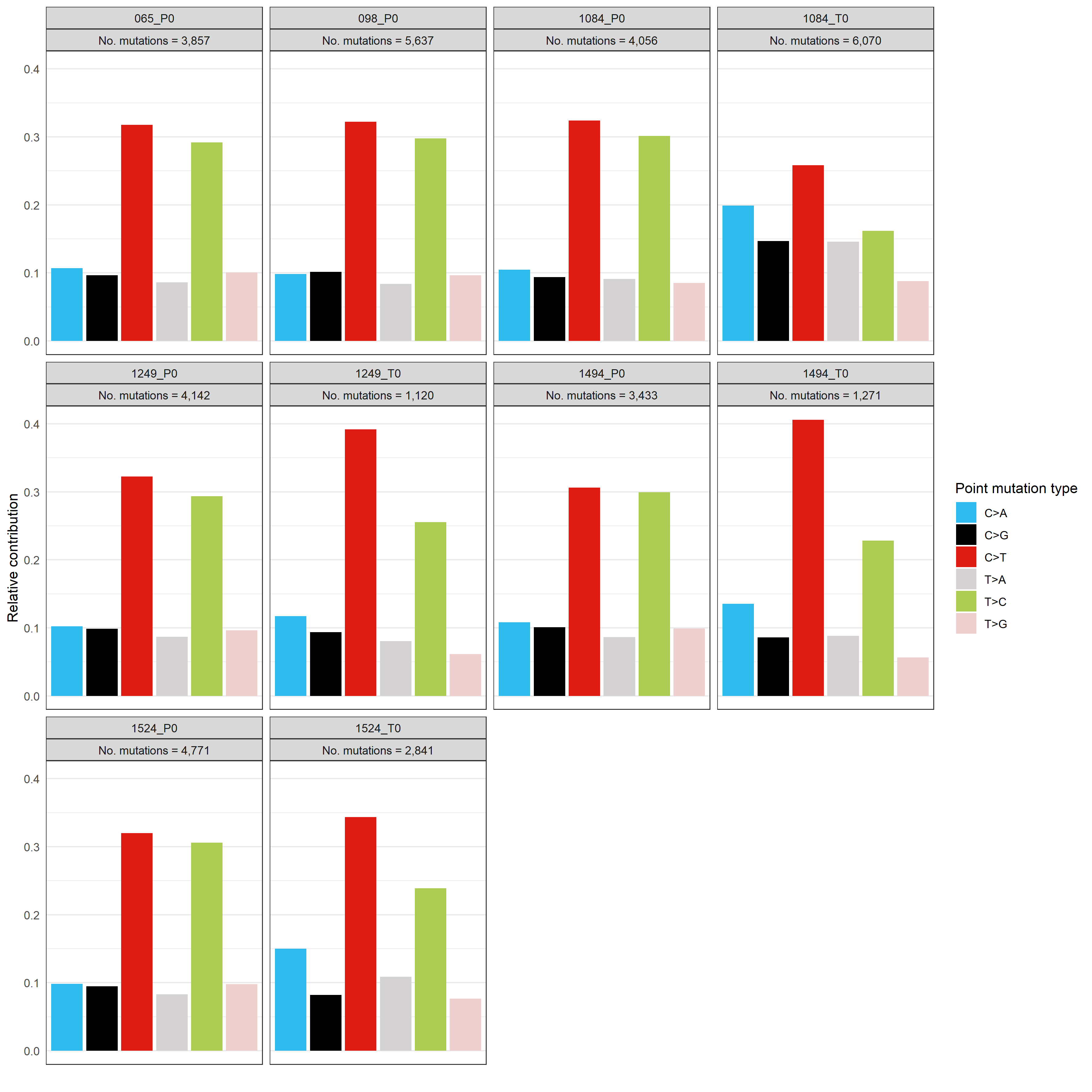


**Supplementary Figure 2:** Relative contribution of each mutation type on all somatic variants detected in plasma and tumour samples. P0 – denotes plasma samples and T0 – denotes tumour samples.


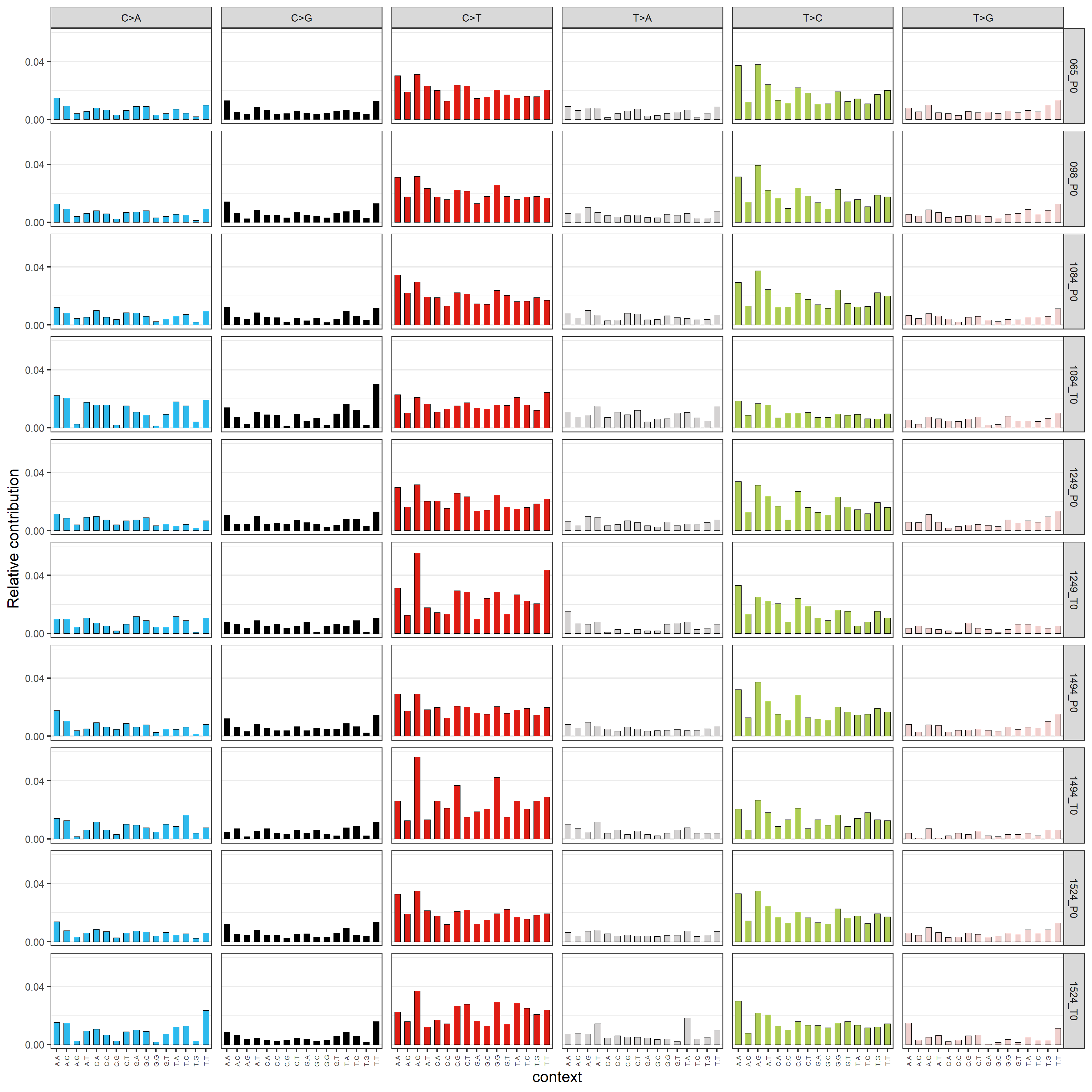


**Supplementary Figure 3:** 96 mutational profile on all somatic variants detected in plasma and tumour samples. P0 – denotes plasma samples and T0 – denotes tumour samples.


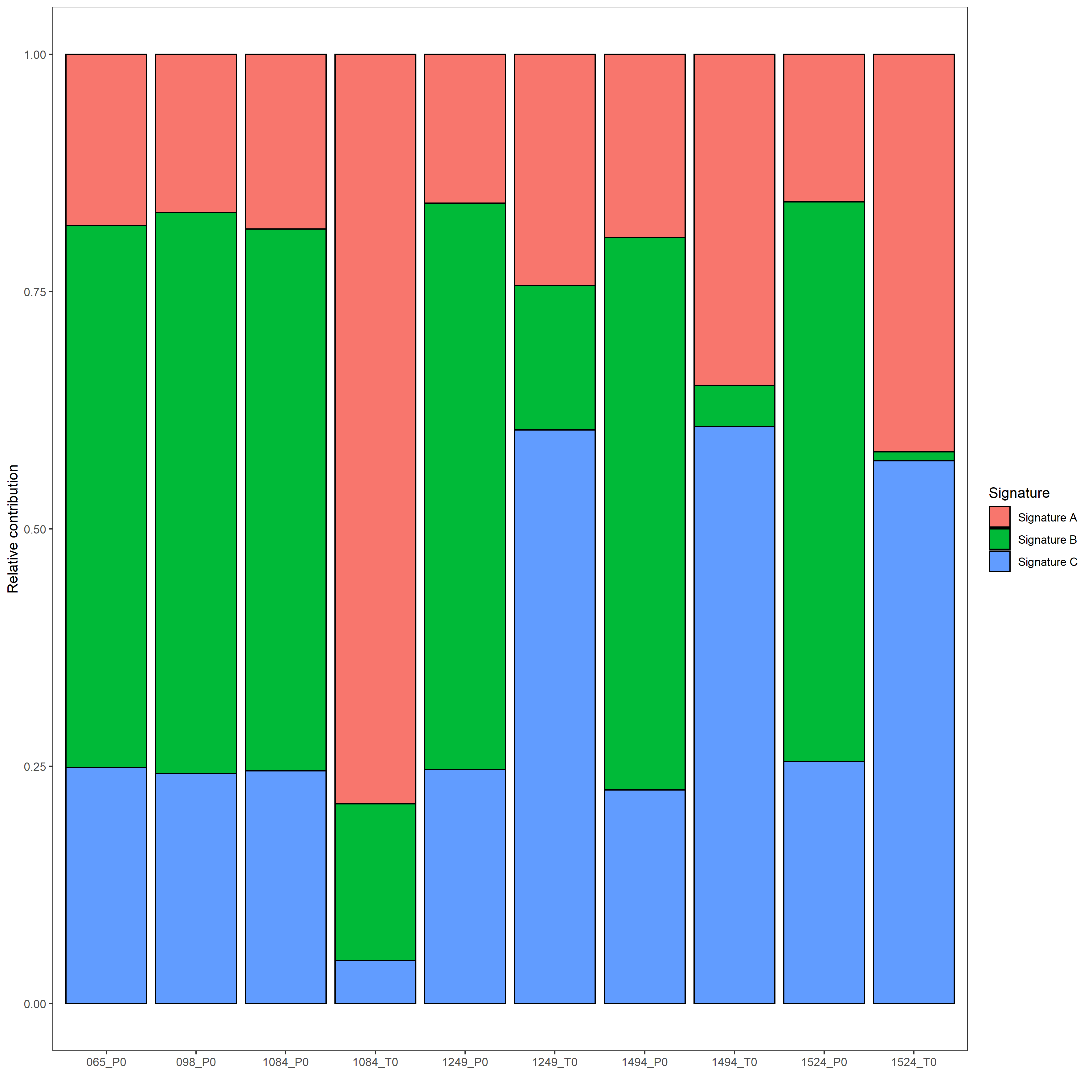


**Supplementary Figure 4:** Relative contribution of extracted mutational signatures in plasma and tumour samples. P0 – denotes plasma samples and T0 – denotes tumour samples.

**Supplementary Table 2:** Cosine Similarity between extracted signatures and COSMIC mutational signatures

| COSMIC Signature | Signature A | Signature B | Signature C |
| --- | --- | --- | --- |
| Signature.1 | 0.45 | 0.50 | 0.79 |
| Signature.2 | 0.38 | 0.16 | 0.35 |
| Signature.3 | **0.91** | 0.71 | 0.55 |
| Signature.4 | 0.72 | 0.40 | 0.38 |
| Signature.5 | **0.80** | **0.91** | **0.84** |
| Signature.6 | 0.44 | 0.53 | 0.71 |
| Signature.7 | 0.34 | 0.28 | 0.46 |
| Signature.8 | **0.88** | 0.58 | 0.55 |
| Signature.9 | 0.61 | 0.64 | 0.58 |
| Signature.10 | 0.34 | 0.19 | 0.31 |
| Signature.11 | 0.38 | 0.40 | 0.49 |
| Signature.12 | 0.46 | 0.79 | 0.66 |
| Signature.13 | 0.45 | 0.15 | 0.12 |
| Signature.14 | 0.52 | 0.43 | 0.56 |
| Signature.15 | 0.38 | 0.42 | 0.55 |
| Signature.16 | 0.69 | **0.83** | 0.71 |
| Signature.17 | 0.25 | 0.27 | 0.24 |
| Signature.18 | 0.55 | 0.22 | 0.30 |
| Signature.19 | 0.55 | 0.59 | 0.66 |
| Signature.20 | 0.49 | 0.66 | 0.62 |
| Signature.21 | 0.28 | 0.55 | 0.53 |
| Signature.22 | 0.30 | 0.19 | 0.13 |
| Signature.23 | 0.32 | 0.38 | 0.43 |
| Signature.24 | 0.57 | 0.29 | 0.32 |
| Signature.25 | 0.71 | 0.65 | 0.59 |
| Signature.26 | 0.40 | 0.74 | 0.67 |
| Signature.27 | 0.23 | 0.14 | 0.16 |
| Signature.28 | 0.23 | 0.30 | 0.20 |
| Signature.29 | 0.63 | 0.38 | 0.44 |
| Signature.30 | 0.51 | 0.53 | 0.65 |

* Signatures with cosine similarity of >0.80 are highlighted in red in the table.

**Supplementary Table 3:** Cosine Similarity between mutational profiles and COSMIC mutational signatures

| COSMIC Signature | 065_P0 | 098_P0 | 1084_P0 | 1084_T0 | 1249_P0 | 1249_T0 | 1494_P0 | 1494_T0 | 1524_P0 | 1524_T0 |
| --- | --- | --- | --- | --- | --- | --- | --- | --- | --- | --- |
| Signature.1 | 0.61 | 0.63 | 0.62 | 0.53 | 0.63 | 0.73 | 0.59 | **0.81** | 0.62 | 0.69 |
| Signature.3 | 0.74 | 0.74 | 0.73 | **0.91** | 0.73 | 0.65 | 0.76 | 0.65 | 0.73 | 0.73 |
| Signature.4 | 0.47 | 0.46 | 0.47 | 0.68 | 0.47 | 0.44 | 0.48 | 0.48 | 0.45 | 0.54 |
| Signature.5 | **0.94** | **0.94** | **0.94** | **0.89** | **0.93** | **0.86** | **0.94** | **0.85** | **0.94** | **0.91** |
| Signature.6 | 0.59 | 0.62 | 0.62 | 0.51 | 0.62 | 0.65 | 0.59 | 0.76 | 0.60 | 0.63 |
| Signature.7 | 0.35 | 0.36 | 0.36 | 0.36 | 0.37 | 0.43 | 0.37 | 0.42 | 0.35 | 0.46 |
| Signature.8 | 0.66 | 0.64 | 0.65 | **0.85** | 0.65 | 0.63 | 0.65 | 0.63 | 0.64 | 0.72 |
| Signature.9 | 0.67 | 0.66 | 0.65 | 0.66 | 0.65 | 0.58 | 0.67 | 0.55 | 0.66 | 0.68 |
| Signature.11 | 0.45 | 0.46 | 0.45 | 0.42 | 0.45 | 0.50 | 0.45 | 0.47 | 0.44 | 0.48 |
| Signature.12 | 0.75 | 0.76 | 0.76 | 0.58 | 0.75 | 0.63 | 0.77 | 0.61 | 0.77 | 0.67 |
| Signature.14 | 0.50 | 0.52 | 0.52 | 0.54 | 0.51 | 0.54 | 0.50 | 0.62 | 0.50 | 0.57 |
| Signature.15 | 0.46 | 0.50 | 0.49 | 0.44 | 0.48 | 0.51 | 0.47 | 0.59 | 0.47 | 0.51 |
| Signature.16 | **0.85** | **0.82** | **0.82** | 0.77 | **0.83** | 0.73 | **0.84** | 0.68 | **0.83** | 0.79 |
| Signature.18 | 0.31 | 0.30 | 0.32 | 0.50 | 0.30 | 0.32 | 0.31 | 0.36 | 0.29 | 0.43 |
| Signature.19 | 0.64 | 0.65 | 0.64 | 0.61 | 0.65 | 0.66 | 0.63 | 0.65 | 0.65 | 0.67 |
| Signature.20 | 0.66 | 0.67 | 0.68 | 0.58 | 0.66 | 0.62 | 0.68 | 0.65 | 0.66 | 0.61 |
| Signature.21 | 0.52 | 0.54 | 0.55 | 0.38 | 0.55 | 0.48 | 0.54 | 0.48 | 0.56 | 0.50 |
| Signature.23 | 0.41 | 0.42 | 0.42 | 0.37 | 0.41 | 0.42 | 0.40 | 0.42 | 0.41 | 0.42 |
| Signature.24 | 0.36 | 0.35 | 0.36 | 0.54 | 0.36 | 0.36 | 0.37 | 0.41 | 0.35 | 0.44 |
| Signature.25 | 0.68 | 0.67 | 0.70 | 0.74 | 0.68 | 0.62 | 0.68 | 0.64 | 0.68 | 0.70 |
| Signature.26 | 0.70 | 0.73 | 0.73 | 0.52 | 0.72 | 0.63 | 0.73 | 0.62 | 0.73 | 0.65 |
| Signature.29 | 0.47 | 0.45 | 0.46 | 0.61 | 0.46 | 0.47 | 0.47 | 0.53 | 0.46 | 0.55 |
| Signature.30 | 0.60 | 0.60 | 0.61 | 0.57 | 0.60 | 0.64 | 0.61 | 0.66 | 0.60 | 0.65 |

* Signatures with cosine similarity of >0.80 are highlighted in red in the table. Signatures with cosine similarity of <0.40 for all samples are excluded in the table.


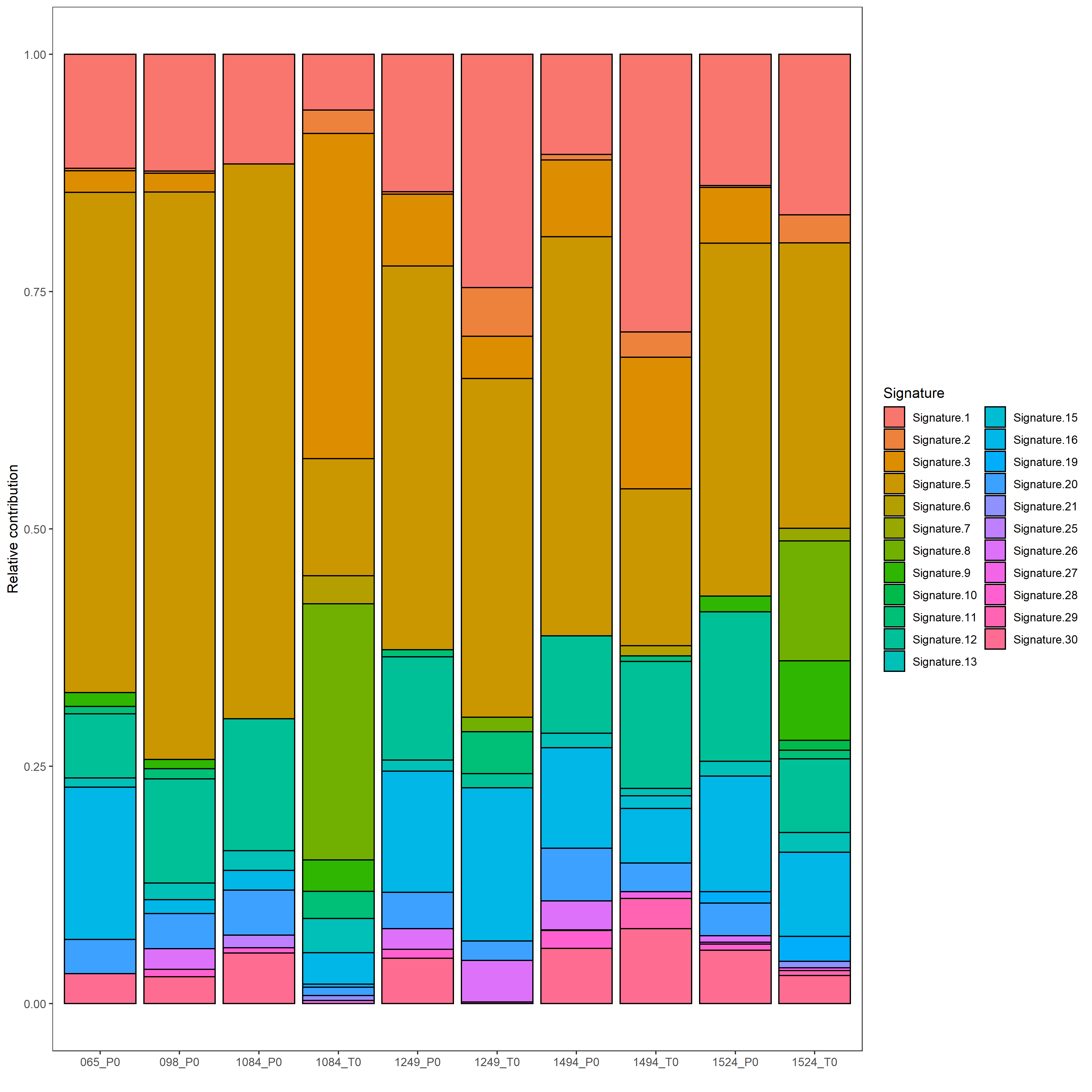


**Supplementary Figure 5:** Relative contribution of COSMIC mutational signatures in plasma and tumour samples. P0 – denotes plasma samples and T0 – denotes tumour samples.


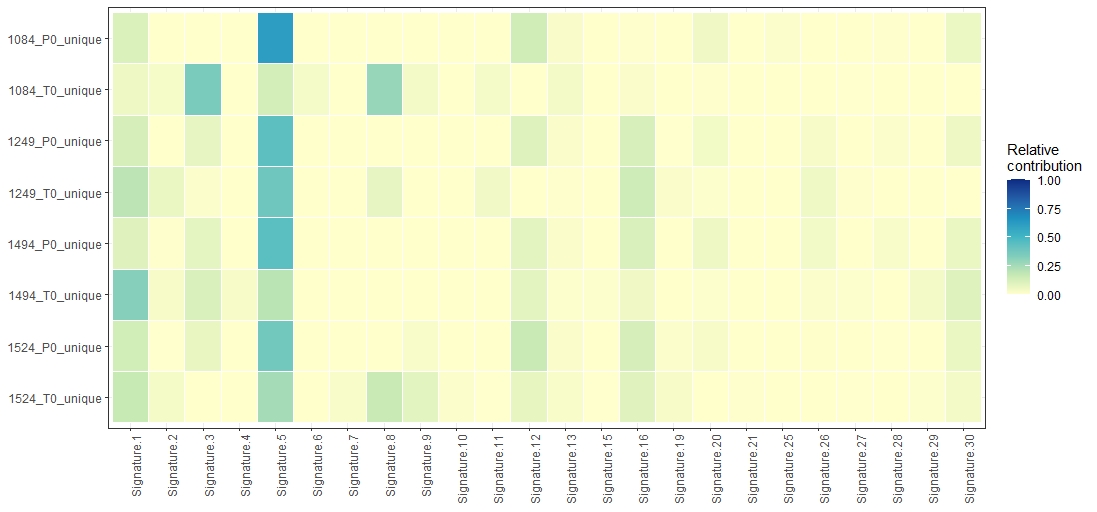


**Supplementary Figure 6:** Relative contribution of COSMIC mutational signatures on unique variants in plasma and tumour samples. P0 – denotes plasma samples and T0 – denotes tumour samples.

**Supplementary Figure 7:** Somatic CNAs detected in patient all tumour and plasma samples. Sample IDs are listed in the plots. Copy number across chromosome 1 to 22 are plotted. The colour of the data points denotes copy number; dark green - 1 copy, blue - 2 copy, brown – 3 copy and red – >4 copy. Light green horizontal line represents a subclonal prediction.


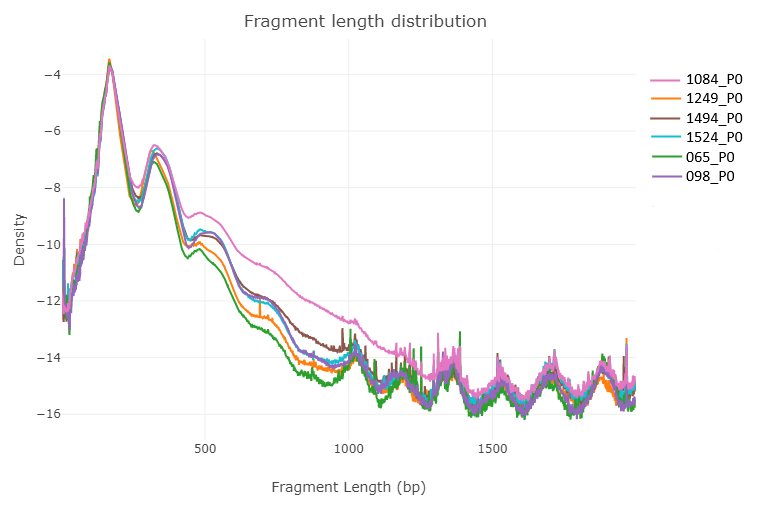


**Supplementary Figure 8:** Cell-free DNA fragment length distribution of Plasma samples for all reads.
